# Annual Meetings of Hospitals

**Published:** 1921-04-16

**Authors:** 


### ANNUAL MEETINGS OF HOSPITALS.

### Leicester Royal Infirmary.

Mr. John E. Faire, J.P., High Sheriff, founder of the "Faire Hospital" which bears his name, a small hospital for paying patients of the middle classes, presided at the 148th anniversary meeting of the Leicester Royal Infirmary held on April 6. Though the Nurses' Recreation Room, where the meeting was held, can seat 200 people, there were many standing. Mr. T. Fielding Johnson, the Chairman of the Board, in moving the adoption of the Annual Report, referred to the great difficulties of the past two years, which began with the removal of wounded soldiers. Financial difficulties have been common to all the voluntary institutions, and have led to much talk about their survival, but now that people are beginning to think more normally there is a strong opinion in favour of maintaining the voluntary system. That opinion is emphasised by Lord Cave's interim report, and he was glad to say it is shared by the subscribers to the Infirmary, and particularly by the contributors to the Saturday Hospital Society, whose contributions amounted to £32,600, approaching double that of the previous year. Notwithstanding the difficulties, the Infirmary's balance-sheet is satisfactory, and therefore no application for a grant was made to the National Relief Fund. The reason for this satisfactory position is that the administration of the hospital thoroughly represents the district it serves, and has the confidence of that district. New departments have recently been opened for the treatment of orthopædic conditions and for pathological work. Many important extensions will follow. The remodelling of the western wing as a memorial to the late Sir Edward Wood has been deferred until the new south-western wing has been erected. When this is complete the institution will have 400 beds. The necessary additions to the nurses' home to provide for the increased number of nurses required have been carried out. The current year is the institution's 150th anniversary year, and the Board hopes to raise sufficient to pay for the new buildings as well as for their maintenance, and to place something to a reserve fund to replace reserves expended.

The High Sheriff, in supporting, referred to the late Mr. T. Fielding Johnson's generous charitable bequests—viz., £1,500 to the Infirmary and Children's Hospital, £1,000 to the Maternity Hospital, and £500 to the Faire Hospital. Thanks were accorded to the Treasurer, Mr. Duncan Henderson, J.P., by Sir Arthur Wheeler, Bart., for his valuable services during the year, and to the honorary medical and surgical staff by Colonel R. F. Martin, C.M.G., and Mrs. Rattray. Dr. Astley Clarke, the senior physician, commented that it was uneconomic and unsound that rising medical men on the staff of hospitals should be struggling with penury, and the position would have to be faced in the near future. In moving the reappointment of the retiring members of the Board the Archdeacon of Leicester remarked that it is because the spirit of voluntary service is foremost in the Infirmary that the institution occupies the position it does. Once this spirit is watered down by the spirit of officialism, voluntary service—the finest thing in the world—will be destroyed.

### Manchester's Children's Hospital.

In the absence of the President, Sir George Agnew, the chair at the annual meeting on April 17 was taken by Mr. Frank H. Roby, Vice-President, who, in bringing before the Governors the particulars of the past year's work, called attention to the fact that, but for the timely help of the National Relief Fund, which had granted £20,000 to the hospital, the financial year would have ended in disaster. Mr. Roby's explanation of the position was but an echo of that given by most chairmen at hospital meetings—namely, that there has been no decrease in the income, but that the expenses have been doubled. In the case of the Children's Hospital the Board of Management very carefully watch over expenditure to prevent extravagance, and it is claimed that, according to all statistics available, the hospital is one of the most economical children's hospitals in England.

It was stated that in some other cities far more is given by the working classes in aid of the hospitals than is given in the Manchester district. This, it is believed, is the fault of insufficient propaganda, as the working classes, in proportion to their position, are most generous towards all charitable schemes which are properly laid before them. Mr. Roby also thought that if business firms were better informed of what was being done for their employees much more support would be forthcoming.

He announced that at the suggestion of the Board of the Children's Hospital a movement had been started to combine all hospitals in the Manchester district for the purpose of appealing to employers and employees. The object was to save energy and waste by united, concentrated action, besides preventing that distraction which results to a would-be subscriber when he receives so many conflicting and scattered appeals.

### Royal Victoria Hospital, Belfast.

Lady Pirrie presided at the annual meeting of the Royal Victoria Hospital, Belfast, on April 1. The annual report for 1920 showed that that year was probably the most difficult and anxious ever experienced during the 128 years of the hospital's existence. The total revenue received from all sources for 1920 was £55,716, an increase as compared with 1919 of £15,669. The total disbursement was £56,796, being higher by £11,466 than in the previous year. Workpeople had increased their contributions, and all other branches of revenue were augmented. As there were deficits in 1918 and 1919, the total indebtedness was £6,830, but the Board of Management expressed themselves as thankful that the position was not worse. Lady Pirrie announced that she had received a cheque from Mr. H. G. Musgrave for £1,000 towards the general fund. She said that in her opinion, if people only realised the work that a hospital did—the procession, pathetic and unending of stricken mortals entering the doors week by week, to issue forth again healed and restored, to take up their normal life—the hospitals would not appeal in vain for the extra funds necessary to carry on such beneficent work.

